# Integrating linkage and radiation hybrid mapping data for bovine chromosome 15

**DOI:** 10.1186/1471-2164-5-77

**Published:** 2004-10-08

**Authors:** Warren M Snelling, Mathieu Gautier, John W Keele, Timothy PL Smith, Roger T Stone, Gregory P Harhay, Gary L Bennett, Naoya Ihara, Akiko Takasuga, Haruko Takeda, Yoshikazu Sugimoto, André Eggen

**Affiliations:** 1 USDA, ARS, U.S. Meat Animal Research Center, Spur 18D, Clay Center, Nebraska 68933-0166, USA; 2 Biochemical Genetics and Cytogenetics Unit, Department of Animal Genetics, Laboratory of Genetics and Biochemistry, INRA-CRJ 78350 Jouy-en-Josas, France; 3 Shirakawa Institute of Animal Genetics, Livestock Technology Association of Japan, Fukushima, Japan

## Abstract

**Background:**

Bovine chromosome (BTA) 15 contains a quantitative trait loci (QTL) for meat tenderness, as well as several breaks in synteny with human chromosome (HSA) 11. Both linkage and radiation hybrid (RH) maps of BTA 15 are available, but the linkage map lacks gene-specific markers needed to identify genes underlying the QTL, and the gene-rich RH map lacks associations with marker genotypes needed to define the QTL. Integrating the maps will provide information to further explore the QTL as well as refine the comparative map between BTA 15 and HSA 11. A recently developed approach to integrating linkage and RH maps uses both linkage and RH data to resolve a consensus marker order, rather than aligning independently constructed maps. Automated map construction procedures employing this maximum-likelihood approach were developed to integrate BTA RH and linkage data, and establish comparative positions of BTA 15 markers with HSA 11 homologs.

**Results:**

The integrated BTA 15 map represents 145 markers; 42 shared by both data sets, 36 unique to the linkage data and 67 unique to RH data. Sequence alignment yielded comparative positions for 77 bovine markers with homologs on HSA 11. The map covers approximately 32% of HSA 11 sequence in five segments of conserved synteny, another 15% of HSA 11 is shared with BTA 29. Bovine and human order are consistent in portions of the syntenic segments, but some rearrangement is apparent. Comparative positions of gene markers near the meat tenderness QTL indicate the region includes separate segments of HSA 11. The two microsatellite markers flanking the QTL peak are between defined syntenic segments.

**Conclusions:**

Combining data to construct an integrated map not only consolidates information from different sources onto a single map, but information contributed from each data set increases the accuracy of the map. Comparison of bovine maps with well annotated human sequence can provide useful information about genes near mapped bovine markers, but bovine gene order may be different than human. Procedures to connect genetic and physical mapping data, build integrated maps for livestock species, and connect those maps to more fully annotated sequence can be automated, facilitating the maintenance of up-to-date maps, and providing a valuable tool to further explore genetic variation in livestock.

## Background

Genome maps for livestock species are necessary to identify genes affecting economically important production traits. Linkage maps, based primarily on highly polymorphic, anonymous microsatellite markers, have been important for identifying chromosomal regions influencing economically important traits in cattle [1-3]. Because a lack of recombination between closely linked markers limits resolution, and because cattle linkage maps [4,5] contain few genes, linkage maps are of limited value for ordering closely linked markers and identifying genes underlying quantitative trait loci (QTL). The radiation hybrid (RH) approach allows mapping monomorphic markers for genes and can provide a higher resolution for ordering close markers [6,7], but high breakage frequency RH data are less reliable than linkage data for ordering widely separated groups of markers [8]. Integrating linkage and RH data into a single map will refine marker order to facilitate genomic sequencing and will also increase the efficiency of identifying genes associated with QTL.

Integrated analysis of both linkage data and RH data allows each source of information to complement the other, providing coarse to intermediate scale maps of the bovine genome, populated with gene markers to facilitate discovery of positional candidate genes for QTL. These integrated maps will lack the fine scale of complete genome sequence, but represent a resource useful for gene identification through comparative mapping approaches, using more complete genome sequence and annotation from other organisms. Similarity between segments of bovine DNA and genomic sequence from other species may supplement integrated data to predict the location of unmapped genes in the bovine genome [9]. A comprehensive integrated map, containing all identified genes and markers, will simplify database queries and reduce ambiguity inherent in mining information from other mammals.

An integrated map can also provide a framework for assembling bovine genomic sequence as data becomes available. A well-ordered map of sequence-tagged-sites (STS) was essential for assembling the human sequence [10]. The National Institutes of Health (NIH) identified the bovine genome as high priority for sequencing [11], and sequencing is underway. One pivotal criterion to classifying the bovine genome as ready for sequencing was the availability of well-maintained genetic and physical maps; integrating these maps will provide additional support for sequence assembly.

Integration of linkage and RH maps has been reported for a number of species [11-14] and individual bovine chromosomes [15-17]. The general approach to integrated mapping has been to score several markers from linkage maps on an RH panel, then align the independent maps via common markers. Nadkarni [18] and White et al. [19] described procedures to synthesize information from multiple independent maps onto a single merged map. These approaches do not directly use data contributing to each map, but merge results of independent analyses. A fundamentally different approach is to merge independent data sets with common markers, so each data set contributes to constructing a single integrated map. Agarwala et al. [20] developed procedures for integrating RH maps where markers common to independent RH panels contributed to the solution of a comprehensive RH map. Schiex et al. [8], developed procedures and released CarthaGene software [21] to merge and solve integrated maps representing multiple linkage and RH data sets.

A large volume of data are being generated in cattle and other livestock species that is not rapidly reflected in current map representations. The result is a lack of truly up-to-date maps of any livestock species, as the maps may lag by months or years in their representation of existing data. It is not feasible to devote significant human resources to constantly maintain and update these maps, so it is critical that automated procedures be developed to free human map curators from many of the time-consuming, error-prone tasks experienced in the mapping process. Existing map construction software is automated to the extent that the likelihoods of many alternative marker orders can be evaluated with a single command, but the entire process of gathering and formatting raw data, constructing maps, examining results and publishing on the internet, or elsewhere, requires human intervention at several stages. Automated procedures will streamline the process in order to focus human effort on the critical stages of verifying raw data and examining the resulting maps.

Bovine chromosome (BTA) 15 provides an interesting example to study the integration of linkage and RH data, and comparison of the bovine to the human genome. A QTL for meat tenderness has been reported on bovine chromosome 15 [22,23]. Comparative mapping indicates that alternating segments of human chromosome (HSA) 11 are conserved on BTA 15 and BTA 29 [15,23,24]. We combined the available linkage and RH data to further examine BTA 15. An integrated linkage and RH map was constructed using CarthaGene software (version 0.99 [21]), and the comparative positions of DNA sequences shared by segments of HSA 11 and the integrated BTA 15 map were established. We also assessed the potential for automating integrated mapping procedures, anticipating a need to extend integration to the entire bovine genome in order to provide up-to-date maps.

## Results and discussion

**Figure 1:**
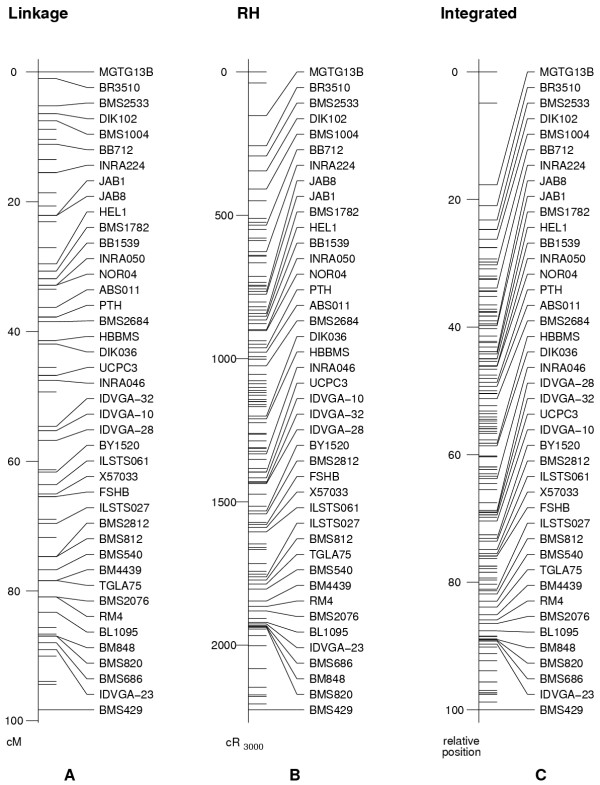
Linear representations of bovine chromosome 15 (BTA15) linkage (A), radiation hybrid (RH; B) and integrated linkage/RH maps (C). Named markers are common to both linkage and RH data sets. Tick marks without a marker name represent markers unique to an individual data set. The linkage map was solved with CRIMAP, and the RH map solved using Carthagene diploid RH data. The integrated linkage/RH map was ordered using CarthaGene with backcross linkage data merged by order with RH data.

The low resolution of the bovine linkage map is indicated by multiple markers sharing the same map position, even when they may be separated by a substantial physical distance. Inclusion of RH data provides additional evidence by which markers that are inseparable only with linkage data can be ordered. The BTA 15 linkage map (Figure 1A; Additional file 1) shows 78 markers placed in 54 distinct positions, with ten positions representing a pair of markers and seven representing three markers. Marker separation on the higher resolution RH map is greater (Figure 1B; Additional file 1), with 109 markers mapped to 105 distinct positions. Projected onto a common scale, the integrated map represents 145 markers in 118 different positions (Figure 1C; Additional file 1). Eighteen positions contain two markers, at three positions three markers are represented, and one position is occupied by four markers.

### Integrated RH and linkage maps

Markers common to both the linkage and RH data sets provide a basis for integrating the data and constructing maps representing both types of data. Primer sequences associated with the RH and linkage markers indicated 42 common markers in the two data sets, with 36 markers unique to the linkage data and 67 unique to RH, for a total of 145 markers represented on the integrated linkage-RH map.

Four sets of markers with different primer sequences matching the same bovine sequence were identified. In two instances (MB064 and HBBMS matching Genbank accession AC130787; T608B5 and SP608B5 matching Genbank accession NM_001752), markers in the set were placed adjacent to each other by the map building routine. In the two other cases (FSHB, FSHBMS, and CSPS101 matching accession Genbank M83753; NCAM1MS and MB085 matching Genbank accession X16451), markers in the set were separated by several markers after initial map construction. In both cases, the map could be reordered so markers in each set were placed next to each other without decreasing likelihood of the map. The final integrated order includes these manual adjustments, so that in all cases of different markers matching the same sequence, the markers are adjacent on the map.

**Figure 2:**
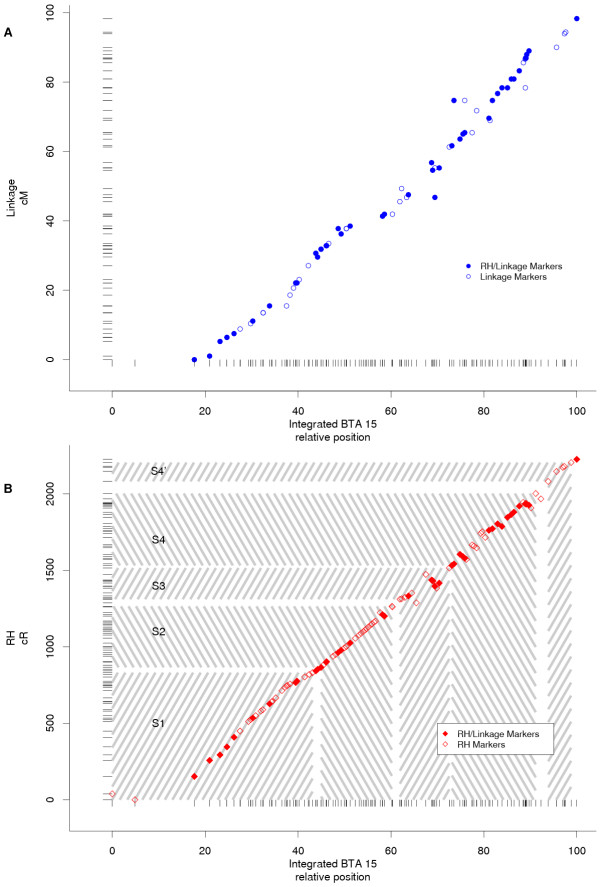
Comparison of independent bovine chromosome 15 (BTA15) linkage (A) and radiation hybird (RH; B) maps with the integrated BTA15 map. The independent linkage map was solved with CRIMAP, and the independent RH map solved using Carthagene diploid RH data. The integrated linkage/RH map was ordered using CarthaGene with backcross linkage data merged by order with RH data. Tick marks along each axis represent positions of markers on the respective linear map. Symbols indicate the intersection of the maps. Symbols forming a straight line indicate agreement between the maps, while deviations from a straight line indicate inconsistencies between the maps. Syntenic group segments are indicated by shading on the RH map (B).

Comparison of the integrated map to independently solved linkage (Figure 2A) and RH (Figure 2B) maps indicates relatively good agreement between the maps. Product-moment correlations between independent (CRIMAP linkage map; CarthaGene diploid RH map) and integrated (CarthaGene backcross linkage data merged by order with diploid RH data) map positions were greater than 0.99 for both the linkage and RH maps. The final integrated map did suggest some rearrangement of both the linkage and RH maps. Solved using CRIMAP, the integrated map order of linkage markers was somewhat more likely than the order of the independent linkage map (lod score of 2.4 favors integrated order). This result suggests that the most likely order identified by the integrated mapping process had not been evaluated while using CRIMAP to construct the linkage map. Because of differences in speed, CarthaGene can feasibly evaluate many more orders than CRIMAP; even without integration with RH data, CarthaGene might be utilized to identify errors in marker order and refine linkage maps.

Comparison of the integrated map to the RH map shows the markers remained in the five blocks identified by Gautier et al. [24], and the order of those blocks is the same for both maps (Figure 2B). Some markers were reordered within blocks of the RH map. As with the linkage map, the integrated map order was more likely than the original independent map order (lod score of 3.4 favors integrated order; both likelihoods solved using CarthaGene with a diploid RH model).

### Comparative bovine and human map

**Figure 3:**
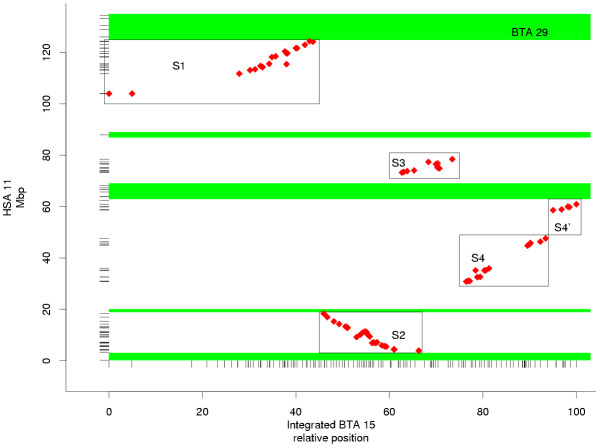
Comparison of the integrated bovine chromosome 15 (BTA15) map with human chromosome 11 (HSA11) DNA sequence (Build 31). Tick marks along the HSA11 axis indicate positions of HSA11 sequence homologous to bovine sequence mapped to either BTA15 or BTA29. Tick marks along the BTA15 axis indicates positions of markers on BTA15. Shading marks regions shared by HSA11 and BTA29. Boxes indicate syntenic group segments.

Comparative map positions for 77 markers mapped to BTA15 were established using *primersearch *[26] to identify bovine DNA sequence associated with each marker, and subsequent BLASTN against HSA11 contig sequences. Positions of the bovine-human matches were between 4.16 Mbp and 135.59 Mbp on the HSA11 draft sequence (Build 31). Percentage identities of the matches ranged from 83% (475/570 bases) to 100% (1941/1941 bases), with a mean of 93% (449/475 bases). The syntenic group segments (S1, S2, S3, S4 and S4') identified by Gautier et al. [24] were retrieved in the comparison of the integrated BTA15 map with HSA11 (Figure 3). The integrated BTA15 map covers approximately 32% of HSA11. There are eight gaps in coverage containing between 4.2 and 25.6 Mbp of HSA11 sequence. Boundaries of the syntenic segments encompass 36% of the loci on HSA11 (Table 1), not counting the 76 loci within large internal gaps in S1 (7.8 Mbp) and S4 (8.9 Mbp). Some of these gaps in HSA11 coverage are syntenic with BTA29 [15,22,23]. Our current BTA29 linkage map places at least one marker in each of the previously identified segments shared by HSA11 and BTA29, accounting for another 15% of HSA11 sequence. Accounting for segments shared with BTA29 leaves 7 gaps containing from 4.9 to 16.1 Mbp of HSA11 sequence that has not been shown to be homologous to mapped regions of bovine chromosomes 15 and 29, although two of the gaps are located within syntenic segments S1 and S4.

**Table 1:** Loci and gene ontology (GO) annotation of human chromosome 11 (HSA11).

	**HSA11 position (Mb)**		**Number of Loci**	**Loci with GO Term**	**Unique GO Terms**
				
**Segment^a^**	**Start**	**End**			
S1	104.0	124.4	200	70	176
S1 gap	104.0	111.8	44	15	54
S2	3.9	18.3	228	52	135
S3	73.3	78.5	80	27	68
S4	30.8	47.6	101	31	86
S4 gap	35.9	44.8	32	6	19
S4'	58.6	60.9	63	12	31
All syntenic regions			672	192	344
internal gaps removed			596	171	310
Entire chromosome		0.0	1640	433	578
>QTL	16.3	20.3	53	16	46
<QTL	122.4	126.4	63	9	25

^a ^Syntenic group segments S1, S2, S3, S4, S4' identified by Gautier et al. (2002). Gaps are relatively long segments within a syntenic group that do not contain sequence common to HSA11 and bovine chromosome 15 (BTA15). Segments designated >QTL and <QTL are 4 Mb segments of HSA11 centered around syntenic markers defining boundaries of S1 (<QTL) and S2 (>QTL), flanking the BTA15 meat tenderness QTL identified by Keele et al. (1998). All syntenic regions represents the union of S1, S2, S3, S4 and S4'. Entire chromosome includes all loci with a position established on HSA11 sequence.

**Figure 4:**
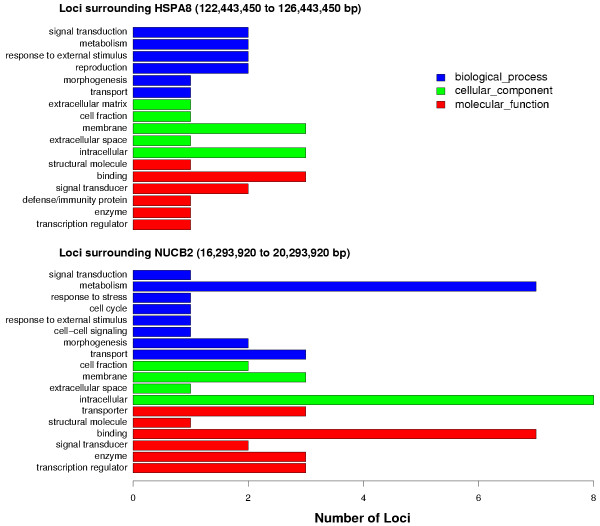
Gene ontology classification of loci on human chromosome 11 (HSA11) in regions near a quantative trait loci (QTL) for meat tenderness. Bovine markers flanking the QTL peak are between defined syntenic regions, so loci in two 4 Mbp regions of HSA 11 (16.3 to 20.3 Mbp; 122.4 to 126.4 Mbp) surrounding markers that define syntenic regions were identified and classified by gene ontology annotation.

Markers more recent [23,24] than the original description of the meat tenderness QTL [22] have resulted in some rearrangment of the BTA15 map, so position of the QTL must be shifted to current positions of markers defining the QTL region. The syntenic segment S1 contains several markers that were within the 95% confidence interval surrounding the QTL, but the two markers most closely flanking the QTL peak, HEL1 and BMS1782, could not be matched to HSA11 sequence and are between defined boundaries of syntenic group segments S1 and S2. Because this QTL region includes a break in bovine-human synteny, the ends of both syntenic segments should be examined to identify positional candidate loci influencing the tenderness QTL. Human loci, in two 4 Mbp segments surrounding the boundaries of S1 and S2 that flank the QTL peak, were identified and associated with gene ontology (GO; [27]) terms to further describe genes near the QTL. These two segments contain 116 loci (Table 1); 25 of these loci have GO annotation [28]) with terms representing various biological processes, cellular components and molecular functions (Figure 4). The GO annotation of loci in both syntenic segments near the QTL may guide further marker development to fine-map the QTL by associations between new markers and tenderness. Adding new markers to this region will also refine boundaries of S1 and S2, and position of the breakpoint between these two segments.

Order is well conserved within syntenic group segments S1, S3, S4' and portions of S2 and S4. The most notable rearrangements within segments are an inversion of several markers in the center of S2, and inconsistent ordering within a subset of S4. The internal rearrangements within syntenic groups found here, pig-human rearrangements [29], and mouse-human rearrangements [30] suggest that precise ordering requires reliable data from the species of interest. Comparative information can be used to predict gene location in regions where within-species mapping data are not available [9] or the available data are ambiguous, and may guide marker development and fine-mapping efforts in specific regions [23,24]. Marker orders based on comparative data, however, should be used with caution. For each systenic segment of BTA 15, marker orders predicted from human order were less likely than the order identified from bovine data (Table 2).

**Table 2:** Comparison of integrated bovine chromosome 15 (BTA15) map where marker order is based on bovine data with alternative maps where segments are reordered according to order of human chromosome 11.

	**log_10_likelihood^b^**			**Total LOD**
		
**Map**	**Linkage**	**RH**	**Total**	
Bovine data	-790.2	-890.4	-1680.6	
Syntenic segments^a ^reordered				
S1	-790.3	-896.5	-1686.8	-6.2
S2	-859.6	-916.4	-1776.0	-95.4
S3	-790.2	-900.0	-1690.2	-9.6
S4	-922.0	-924.6	-1846.6	-166.1
S4'	-790.2	-892.1	-1682.3	-2.3

^a ^syntenic group segments described by Gautier et al. (2002).

^b ^likelihoods computed with backcross linkage data merged by order with diploid RH model data

Challenges for building high-resolution integrated maps and leveraging data from various sources, both within and across species, will be to determine regions where additional data may be informative and placing appropriate emphasis on the different sources of information at different levels of resolution. Linkage maps can provide the scaffold for ordering an entire chromosome, so linkage data may receive the greatest emphasis for initially determining a coarse order. Increased emphasis should be given to higher resolution RH and other physical mapping data to resolve order where placement of linkage markers is uncertain, and markers are too close to provide definitive order. Comparative sequence and mapping information from other species should be most useful to position markers within regions where physical data have insufficient resolution and within-species sequence data are not available. Using appropriate weights to combine genetic and physical mapping data, within-species sequence and comparative sequence data should allow the different data sources to complement each other, resulting in consensus maps supported by the combined sources of information.

### Automation

Genome maps of livestock species need to represent current information in order to maximize utility of the maps. Positions of putative QTL may become misleading if QTL positions are not updated to reflect subtle rearrangements resulting from new mapping data. Genes associated with phenotypic variation will be more readily identified if available information to link mapping data to genes and their function is maintained. Continually updating the maps to depict relevant existing information will be facilitated by automation, but a number of issues must be addressed for implementation of automated procedures to be fruitful.

Access to dynamic sources of mapping data must be maintained, so that new information can be incorporated into the maps soon after it is generated. Information to connect data from various sources must be available to expedite integration. Map computation strategies deserve some attention, to minimize the delay between acquiring new data and appearance of those data in subsequent maps.

Procedures developed to integrate BTA15 linkage and RH data can be applied to available data for the entire bovine genome. The integration effort will be more valuable, however, if sources of data for the integrated map are periodically updated. Success of a comprehensive integration effort will also depend on information available to establish connections between the data sets. One alternative is to resolve marker nomenclature, perhaps by developing and maintaining a database of marker names and synonyms. A more straightforward, and easily automated, approach is to use primer sequences as universal identifiers to establish connections between mapping data sets. Database curation efforts to associate mapping records (animal genotypes and RH vectors) with primer pairs may be more worthwhile than attempts to resolve all possible names for a given marker.

Primer sequence can also be used to establish connections to sequence databases. Sequence similarity searches should reveal connections to STS sequences associated with markers; the process will also identify connections to other sequences, including more completely annotated and assembled sequence. Sequences identified in this process can be used to establish connections with human and other well annotated, assembled genomic sequence for comparative mapping. Similar associations between mapping and sequence data may be established using marker and locus names, provided that marker nomenclature can be resolved Sequence-based connections between mapping data sets, integrated maps, and genomic sequence may be more reliable and are more amenable to automation than attempts to connect sources using names and other information.

Connections between maps and annotated sequence can accelerate positional candidate gene discovery if the sequence annotation includes functional information. Harhay and Keele [31] used GO and GO-annotated human sequence to link livestock EST with function; mapping the EST can extend their procedures to relate map position to function. Connecting map positions to GO terms requires synchronizing several information sources, including livestock maps, human sequence annotated GO terms, and GO databases.

Placement of new markers on integrated maps must keep pace with new marker development, if integrated maps are to remain current with available mapping and sequence data, The basic concept of map construction, finding the most likely marker order out of all possible orders, is conceptually simple but computationally demanding, because the number of possible orders increases factorially with the number of markers. Evaluating all possible marker orders is not feasible when mapping data represents more than twenty or thirty markers on a chromosome. Cost and time constraints limit map construction to strategies that evaluate a sufficient number of possible orders to ensure that a reasonably good order is identified.

As bovine sequence data becomes available, methods to exploit that resource to refine both the integrated maps and sequence assemblies must be implemented. Advent of whole-genome sequence assemblies has not diminished the value of maps in sequenced species. Discrepancies between human maps and sequence assemblies have been noted [32,33], although concordance between a SNP linkage map and sequence assemblies increased in later assemblies [33]. A comprehensive linkage-RH map has been used to validate mouse sequence assemblies, revealing cases of significant inversions and translocations in sequence, as well as confirming sequence order in other regions where the sequence order disagrees with previous mouse RH maps [34]. An integrated linkage-RH map of the rat suggests some errors in the draft sequence, but more importantly, provides a mechanism to anchor QTL on the genomic sequence [35].

The strategies employed must be sufficiently flexible to allow manual manipulation of the resulting maps. Some evidence, such as STS markers sharing the same sequence, and ordering information from other species, is not easily represented in linkage and RH mapping data. In some cases of markers sharing the same sequence, markers can be forced to share the same position, or data from multiple markers can be combined to create a single haplotype representing multiple markers. Marker orders suggested by maps of other species may be compared with likely orders identified from within-species data. Incorporating information not directly represented in mapping data can require manually evaluating additional orders, and making some judgement about which results are most acceptable.

In exploratory analyses merging BTA15 linkage and RH data, simulated annealing and taboo search algorithms in CarthaGene were explored as methods of initially ordering the integrated map, before refinement with the polish and flips routines. Resulting maps were similar to the map presented, but required more than 24 hours to compute. The map presented was initially ordered by placing each marker against a pair of markers common to both data sets, and was constructed in less than four hours. Another approach involved initially placing markers against the set of all markers common to both the linkage and RH data sets, in the linkage map order. While map construction was somewhat faster using this approach, the resulting map was less likely than the map initiated from a pair of markers and showed greater disagreement with the linkage map.

Parallelization of the mapping algorithms can substantially increase the speed of map construction. Likelihoods of a number of alternative orders must be computed at several steps during the map building process. If these calculations are distributed across multiple processors, time required to compute all likelihoods and arrive at a final order will be reduced because computations are performed simultaneously. Increased parallelization should also increase the feasibility of implementing more thorough algorithms that examine a larger number of possible orders, therefore increasing the probability of identifying more likely maps.

## Conclusions

Linkage and radiation hybrid maps are powerful tools to facilitate discovery of genomic regions and ultimately genes influencing livestock production traits. Combining linkage and RH data can provide more accurate, consolidated maps representing more information, especially if the maps are connected to well annotated genomic sequence. Automating map construction and comparative mapping procedures will expedite construction of whole-genome integrated maps and maintaining a comprehensive resource as new data becomes available. Success of automated procedures to connect data from various sources and construct integrated maps depends on information available to establish connections between data; sequence-based approaches to connect data are preferrable.

## Methods

### Data sets for integrated map construction

Linkage data for 78 markers in the BTA15 linkage group were obtained from the U.S. Meat Animal Research Center (MARC) reference population (224 animals; [4]). Radiation hybrid data for 109 markers were obtained from the ComRad project radiation hybrid panel (94 cell lines; [7,24]). These data include two newly developed microsatellite markers genotyped in the MARC families (Table 3), and seventeen previously unpublished markers with RH data(Table 4).

**Table 3:** Description of previously unpublished linkage markers placed on the integrated bovine chromosome 15 map.

**Marker Name**	**Forward Primer**	**Accession number**
	**Reverse Primer**	
DIK2411	CTAACGCCCCTGAGACAGAC	AB112806
	GTGGCGTTAGTTGGTCCTTC	
DIK2374	CCTGTTTGGGACACTCTCCT	AB112803
	GAATCTCTTCAATGCCGAATG	

**Table 4:** Description of previously unpublished radiation hybrid markers placed on the integrated bovine chromosome 15 map.

**Locus Symbol**	**Gene Name**	**Forward Primer **	**Accession Number**
		**Reverse Primer**	
C11ORF15	chromosome 11 open reading frame 15	GCATCCTAGAACAGACTGGCT	AW657178
		GGAGGCAACCGGAACTCCAGT	
DKK3	dickkopf (Xenopus laevis) homolog 3	CGAAGACCATTATCAGCCACA	AW336328
		CTCTGGATGCATACATGAAGGA	
EIF4G2	eukaryotic translation initiation factor 4 gamma, 2	AGCTTGAGGCCTGCTCAGTCT	AV602677
		GTCCCAAAGGTGGCGTTTGA	
FLJ11790	protocadherin 16 dachsous-like (Drosophila)	CCCAGCTTCTCACCTTCACTA	AW428073
		GATATGGAGCTCGGTGTCGTCT	
INPPL1	inositol polyphosphate phosphatase-like 1	CAGCTCAACTTGGAGCGGGAA	BF705795
		GAACCCCGCTCATAGCGGTAA	
KIAA0750	hypothetical protein KIAA0750	GTGGGAAGCTGGCTATTGCA	AW652984
		GAAGATGAAAGCCACACCGCT	
MRPL17	mitochondrial ribosomal protein L17	CACCTGTTGCAGAACTTGCTT	BE899833
		CCCAGCTTCCCGTAGTCAATA	
PARVA	parvin, alpha	GCCGTATCCCTCAACTCCTTT	BE477207
		CTCAAGAGTCCCTGTTGAAGA	
PSMA1	proteasome (prosome, macropain) subunit, alpha type, 1	GAATATGCAATGGAAGCTGTC	AV602233
		GCTGCAAGTTCTGACTGTGCT	
RANBP7	RAN binding protein 7	GGGTGAAGAGATGAGGAAGAT	BF45355
		CTGATACTCATCAACAGGGTT	
RNF21	tripartite motif-containing 34	GAAGAGAAACTCCTACTCTTCT	AW447003
		CTCCTGAGATCGTTCACAAAGA	
ST5	suppression of tumorigenicity 5	CGCTGCTCTGGTCTATCACTT	BF604586
		ATTGCCAGCCCCTGGCAGGAA	
STIM1	stromal interaction molecule 1	GCCCTCCAGGCTAGCCGAAAT	BE756550
		CACTGCCACCCCCATCCTGTT	
TAF2H	TAF10 RNA polymerase II, TATA box binding protein (TBP)-associated factor, 30 kDa	TGGTGTCCAGCACGCCTCTA	AW315164
		GTAGTAACCAGTCACTGCATCA	
UVRAG	UV radiation resistance associated gene	GTACATTTTCAGCTGAGCACC	BE590188
		CGCGGTACACTCCTTTCTCAA	
WEE1	wee1+ (S. pombe) homolog	GATGGATGCGTTTATGCCATA	AV598317
		CGAACTACATGAGAATGTTGC	
ZFP26	C3HC4-like zinc finger protein	CTGCTAAAGTGGCTTCTGGC	BF04414
		GGTACAGACCACTCGTACAA	

All bovine sequence information stored in GenBank was identified using the taxonomy ID field of the sequence file annotation and obtained from NCBI. Provisional sequence data consisting of tentative consensus clustering of bovine EST data was obtained from the *Bos taurus *gene index (BTGI; [36]) assembled by The Institute for Genonomics Research (TIGR, [37]). Other sources of sequence were the NCBI nt database (NT; [38]), and human chromosome 11 draft sequence contigs (Build 31;[38]).

### Data integration

Connections between data sets are necessary for integrated analyses of those data to be meaningful. Because some marker names were ambiguous, connections between markers in the linkage and RH data were established using primer sequence. Markers with identical primers were considered to be the same, regardless of marker name. Primer sequence was also used to establish connections with human sequence. Primer pairs were matched against bovine sequence from GenBank, NT and BTGI databases using the EMBOSS [26]*primersearch *tool. The longest matching sequence having one or fewer mismatches and an amplimer less than 600 bp was selected for homology search against HSA11 contigs. The selected sequences were examined for gaps, and where gaps occurred, only the ungapped pieces matching a primer pair was used in the homology search. Connections between the individual sequences matching bovine markers and human sequence were then determined via BLASTN [39] with an expectation value of e^-20^, and default values for other parameters.

Connections between human position and functional GO annotation were extracted from the downloadable LocusLink database [38]. Procedures using the GO database [40] and perl API [41] were developed to classify specific GO terms into general categories described by higher level terms.

### Integrated map construction

Observations for RH data are binary (0/1), indicating absence or presence of a particular marker in a cell line, where each cell line represents a relatively short segment of DNA on a chromosome. Physically close markers are more likely to be observed on the same cell line than distant markers. Linkage data includes pedigree information and marker genotypes, where individual genotypes represent alleles inherited from each parent. Alleles for physically close markers on a single chromosome are more likely to be inherited from the same grandparent; the likelihood of marker alleles with different grandparental origin appearing on the same chromosome increases with distance between markers. These chromosomes can be represented in a binary, RH-like format that can be merged with RH data using CarthaGene. Analagous to RH data representing presence or absence of a marker in a cell line, binary representation of linkage data indicates presence or absence of a maternal allele on an individual chromosome. The *chrompic *option of CRIMAP [42] was used to construct these individual chromosomes, using the most likely order identified by an automated linkage mapping routine. No distinction was made between definite phase-known maternal and paternal inheritance, and statistically predicted inheritance when phase could not be determined.

An interface to the CarthaGene shared library was developed using perl and the perl Inline modules [43] to automate map construction (see Additional file 2). This interface includes procedures to initially place markers on a map and refine map order, as well as a number of utility routines. A map construction script using this interface was also developed (see Additional file 3). The script to order markers on the integrated map starts by merging the binary backcross representation of linkage data with the haploid model RH data, assuming common marker order (*dsmergor*). Two markers shared by the linkage and RH data sets are identified, and all other markers are inserted, one at a time, into the most likely position using the CarthaGene *buildfw *procedure. Once all markers from both data sets are placed, the marker order is refined iteratively, cycling through *polish *and *flips *routines until likelihood does not improve. The *polish *procedure individually tests each marker in all alternative positions, and *flips *evaluates permutations of all sets of six adjacent markers.

After convergence using the map construction script, further evaluation of alternative orders was carried out with the backcross linkage data merged with a diploid model of the RH data, again assuming common marker order. Marker orders consistent with available sequence information were evaluated. Where primer paris for different markers matched the same bovine sequence, but the markers were separated by one or more other markers by the map construction routine, likelihoods of orders with the matching markers placed adjacent to each other were determined. Likelihoods of marker orders consistent with human sequence within each syntenic segment were also computed. The sequence-based orders were used in the final integrated map if they did not decrease likelihood of the map. Log-likelihoods of the final integrated map order were computed with the RH and linkage data sets for comparison to the independent maps, using CarthaGene for the RH map and CRIMAP for the linkage map.

The final integrated marker order was projected onto a common relative scale representing all markers. This was accomplished by merging the linkage data with RH data, modeled as backcross, using *dsmergen*. Marker order was set to the final integrated order, map distances computed, then scaled to range from zero to 100.

### Computation

All computation was performed on a 10-node Linux cluster, each node configured with 2 AMD 1900+ CPUs and 3 Gb RAM. When practical, computation was parallelized using perl scripts and open source Grid Engine software [44] to distribute tasks to each node in the cluster. Steps that were parallelized included matching primers to sequence, and the Blast searches to align bovine with human sequence.

## Authors' contributions

WMS developed perl scripts for automated analyses, conducted analyses to match markers from linkage and RH data sets, constructed the linkage and integrated maps, and drafted the manuscript. MG and AE developed RH markers and constructed the RH map. AE and JWK conceived the research, and contributed to planning analyses and evaluating results. RTS and TPLS assisted with evaluation of the integrated map. GPH conducted BLAST analyses and associated bovine sequence with human GO annotation. NI, AT, HT developed linkage markers. YS and GLB coordinated linkage map data collection. GLB curates the MARC linkage data and linkage maps.

## Supplementary Material

Additional File 1 linkage, RH and integrated maps of BTA 15 Click here for file

Additional File 2 perl interface to CarthaGene, requires perl Inline::Tcl module Click here for file

Additional File 3 perl script to construct integrated maps using CarthaGene, requires carthaPerl.pl interface to CarthaGene Click here for file
